# Does Habitat Variability Really Promote Metabolic Network Modularity?

**DOI:** 10.1371/journal.pone.0061348

**Published:** 2013-04-12

**Authors:** Kazuhiro Takemoto

**Affiliations:** 1 Department of Bioscience and Bioinformatics, Kyushu Institute of Technology, Kawazu, Iizuka Fukuoka, Japan; 2 PRESTO, Japan Science and Technology Agency, Kawaguchi, Saitama, Japan; UMIT, Austria

## Abstract

The hypothesis that variability in natural habitats promotes modular organization is widely accepted for cellular networks. However, results of some data analyses and theoretical studies have begun to cast doubt on the impact of habitat variability on modularity in metabolic networks. Therefore, we re-evaluated this hypothesis using statistical data analysis and current metabolic information. We were unable to conclude that an increase in modularity was the result of habitat variability. Although horizontal gene transfer was also considered because it may contribute for survival in a variety of environments, closely related to habitat variability, and is known to be positively correlated with network modularity, such a positive correlation was not concluded in the latest version of metabolic networks. Furthermore, we demonstrated that the previously observed increase in network modularity due to habitat variability and horizontal gene transfer was probably due to a lack of available data on metabolic reactions. Instead, we determined that modularity in metabolic networks is dependent on species growth conditions. These results may not entirely discount the impact of habitat variability and horizontal gene transfer. Rather, they highlight the need for a more suitable definition of habitat variability and a more careful examination of relationships of the network modularity with horizontal gene transfer, habitats, and environments.

## Introduction

Because of the importance of modular organization in biological systems [1], modularity in cellular networks is of great interest to researchers of basic science as well as to those in engineering, in the context of *network biology* [2], [3]. Modularity is an especially important property because it is related to robustness [4] and evolvability [5]. Nonetheless, skepticism exists regarding the importance of modularity [6], [7].

The origins of network modularity have been of particular interest to researchers. Kashtan and Alon [8] have suggested a possible theoretical model that uses an evolutionary optimization algorithm based on edge rewiring (mutation). This theory is based on the conjecture that modular networks spontaneously evolve when the evolutionary goal (i.e., system-specific purpose) changes over time in a manner that preserves the same subgoals but in different permutations. Similarly, Lipson et al. [9] suggested that evolutionary forces can lead to modularity. In this context, for example, an evolutionary goal can be interpreted as survival of a species in a natural habitat; thus, a change in the evolutionary goal corresponds to the variability in a species' habitat.

Inspired by these studies, Parter et al. [10] showed by using network analysis that variability in natural habitats promotes the modularity in bacterial metabolic networks (i.e., network modularity in organisms increases with increasing environmental variability). These researchers focused on metabolic networks because these networks are believed to be highly modularized [11], [12]; however, it has also been suggested that metabolic networks are modular but not significantly so [13]. A diversity of species' metabolic networks are available in databases such as the Kyoto Encyclopedia of Genes and Genomes (KEGG) database [14] and the Encyclopedia of Metabolic Pathways (MetaCyc) [15] although metabolic information is still not completed. The result shown by Parter et al. clearly supports the predictions from theoretical models, and several studies actively discuss ecological interactions of metabolic networks based on habitat variability (e.g., [16]–[20]).

However, further data analyses have begun to cast doubt on this interpretation of network modularity, which is evidently derived from a viewpoint of evolutionary optimization or habitat variability. For example, the rate of edge rewiring due to evolutionary events is the lowest in cellular networks [21] (although previous theories have assumed the edge rewiring mechanism), suggesting that it is difficult to completely explain the origin of metabolic network modularity on the basis of edge rewiring. In Archaea (another domain of prokaryotes), changes in metabolic network modularity depend on growth conditions such as temperature and trophic requirements, and are not necessarily reliant on habitat variability [22]. Archaea, however, represent an unusual case in that they show high diversity based on growth conditions [23], but not on habitat variability. Similarly, several studies also showed that a species' growth conditions influence its metabolic network structure [24], [25].

Several theoretical studies have questioned the view that network modularity is the result of a change in evolutionary goals. Using a network model, Solé and Valverde [26] claimed that such a mechanism is not required for acquiring network modularity. However, they focused on protein interaction networks, not metabolic networks, and only presented qualitative results on the origin of network modularity. Therefore, we proposed an evolving network model without tuning parameters to describe the metabolic networks, and demonstrated *quantitatively* that metabolic network modularity could arise through simple processes, independent of changes in the evolutionary goal or habitat variability [27].

These findings cast doubt on the impact of habitat variability on modularity in metabolic networks. We re-evaluated this impact by using statistical data analysis and the latest metabolic reaction database.

## Results

### It is difficult to conclude that habitat variability promotes metabolic network modularity

Parter et al. [10] reported a positive correlation between metabolic network modularity and the variability in a natural habitat. In this previous study, the habitat variability was classified into 6 groups according to the NCBI BioProject database (www.ncbi.nlm.nih.gov/bioproject) as follows (see [10] for details): (1) *obligate* bacteria that are obligately associated with a host, either intracellularly or extracellularly, (2) *specialized* bacteria that live in specialized environments such as marine thermal vents, (3) *aquatic* bacteria that live in fresh or seawater environment, and are not associated with hosts, (4) *facultative* bacteria, free-living bacteria that often associate with a host, (5) *multiple* bacteria, that live in multiple different types of environments such as bacteria with a wide host range, and (6) *terrestrial* bacteria that live in the soil. The parenthetic numbers correspond to the degree of habitat variability. We also used this definition of habitat variability in this study.

In recent years, however, several new technologies and high-throughput methods have generated a greater volume of genomic and metabolic data (i.e., metabolic information has been significantly updated). Consequently, it is possible to arrive at different conclusions about correlations between network modularity and habitat variability. Therefore, we reinvestigated the correlation between network modularity and habitat variability by using the latest metabolic reaction database [28]. The definition of habitat variability, the calculation method, and the set of species were similar to those used in the previous study [10]. In this analysis, however, *Methanosarcina acetivorans* and *Agrobacterium tumefaciens* C58 (Cereron)–both of which were used in the previous study–were excluded because the former belongs to a different domain (Archaea) and the latter was not available in the KEGG database as of May 20, 2011. In total, we investigated the metabolic networks of 115 bacteria (see Table S1).

We constructed metabolic networks–represented as undirected networks in which nodes and edges correspond to metabolites and substrate–product relationships, respectively–and calculated their modularity values (*Q*), as described in previous studies [10], [22], [27] (see Materials and Methods for details). Although metabolic networks are obviously directed, we considered undirected networks for comparison to the previous study [10]. Note that the neglect of edge direction does not indicate any importance of edge direction. When considering edge direction, for example, we can find an important structural pattern in metabolic networks: bow–tie structure [29], [30]. Note that the modularity value was normalized (*Q_m_*) to allow comparison between different network sizes and connectivity, parameters that strongly affected this variable [31]. Thus, there was no correlation of *Q_m_* with the number of nodes (i.e., metabolites; Spearman's rank correlation coefficient *r_s_* = 0.057, *p* = 0.54) or the number of edges (i.e., substrate–product relationships in metabolic reactions; *r_s_* = 0.082, *p* = 0.38). Furthermore, *Q_m_* was not correlated with genome size (*r_s_* = 0.054, *p* = 0.56) or with the number of protein-encoding genes (*r_s_* = 0.078, *p* = 0.41), because these variables are related to network size and number of edges (see Table S1).

**Figure 1:**
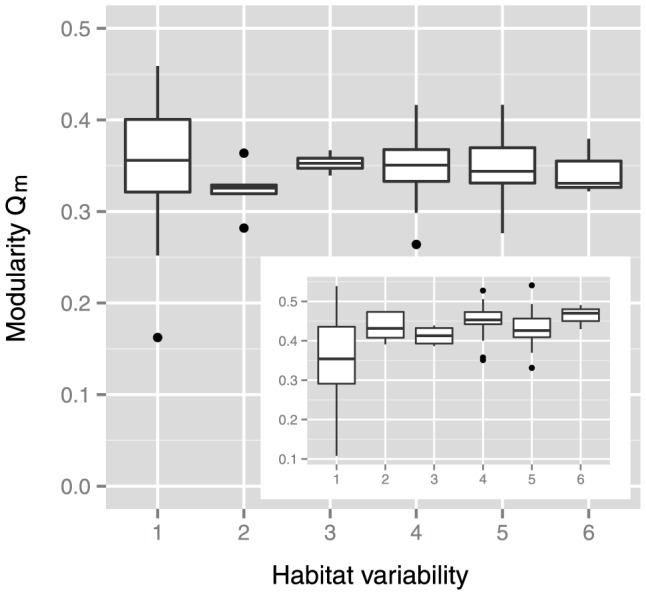
Correlation between network modularity and habitat variability in a natural habitat. Metabolic networks constructed from the latest version of the database show no correlation (*p* = 0.61 using the Kruskal-Wallis (KW) test; Spearman's rank correlation coefficient *r_s_* = −0.03 and *p* = 0.78). Metabolic networks constructed from the early version of the database (inset) demonstrate a positive correlation (*p* = 0.0001 using the KW test; *r_s_* = 0.31 and *p* = 0.0008).

As shown in Figure 1, we could not conclude that a positive correlation existed between modularity and habitat variability in bacterial metabolic networks, as in Archaea [22]. This result does not support the hypothesis that variability in natural habitats promotes modular organization.

We used a fast greedy algorithm [32] to calculate metabolic network modularity (i.e., *Q* and *Q_m_*) (see Material and Methods for details) although this algorithm is known to be poor in finding the maximum *Q* [33]. Thus, it remains possible that the limitation of the fast greedy algorithm causes the conclusion of no correlation (Figure 1). To avoid this limitation, we need to use better algorithms such as the Bayesian method [34], the spectral decomposition method [35], and simulated annealing-based methods [12], [36] (reviewed in [37]); however, these algorithms could not be applied to this study because their higher computational costs. However, the limitation of the fast greedy algorithm posed a little problem for calculating *Q* in this study. We checked the difference of *Q* in each metabolic network between the fast greedy algorithm and a simulated annealing-based method [36] (i.e., *Q*_Greedy_−*Q*_SA_, where *Q*_Greedy_ and *Q*_SA_ are *Q* calculated using the greedy algorithm and simulated annealing-based method, respectively). We found that the mean of the difference is negative (−0.0053; 95% confidence interval, from −0.0058 to −0.0048; see also Table S1), as expected. However, this difference was very small in comparison with the mean of *Q*_Greedy_ or *Q*_SA_ (0.798 and 0.803, respectively) (see also Table S1).

The network modularity *Q_m_* was calculated using the largest connected component metabolic networks (see Material and Methods for details) although metabolic networks are fragmented (i.e., possess isolated components) in general. Thus, the conclusion in this study is limited to the context of largest connected components. However, this handling (i.e., extraction of largest connected components) hardly influences the conclusion. Using the latest version of metabolic networks, we checked the difference of *Q* between the entire network and the largest connected component (i.e., *Q*_Entire_−*Q*_LCC_, where *Q*_Entire_ and *Q*_LCC_ are *Q*s calculated from the entire network and largest connected component, respectively). The mean of the difference was −0.035 (95% confidence interval, from −0.037 to −0.034), indicating that *Q* values of the entire networks are slightly larger than those of the largest connected components because isolated components are identified modules of the entire network. However, this difference was small in comparison with the means of *Q*_Entire_ or *Q*_LCC_ (0.798 and 0.833, respectively) (see also Table S1).

### Lack of data on metabolic reactions may result in an overemphasized role of habitat variability in increasing network modularity

We attempted to perform this re-evaluation under similar conditions as those used in the previous study [10]. However, the conditions for data analysis may have been slightly different from those used in the previous study. For example, the network representation in this study is slightly different from that in the previous study. We defined edges as substrate–product pairs on the basis of carbon trances (see Materials and Methods for details) in this study. This approach was inspired by Arita's study [38], in which he pointed out that the pathways computed in the classical manners (i.e., network representations without consideration of atomic traces) do not conserve their structural moieties and, therefore, do not correspond to biochemical pathways on the traditional metabolic map. On the other hand, the definition of edges in the previous study was merely based on the KEGG database, without explicit consideration of carbon traces.

The definition of currency metabolites such as water and ATP may also have been different from that in the previous study because the definition has not been clearly described in [10]. The deletion of currency metabolites is a crucial step for metabolic network analysis from a topological point of view [39], [40].

To show that differences in analytical conditions pose few problems, we performed similar analyses by using the earlier version of the metabolic reaction database [39]. As shown in the inset of Figure 1, a positive correlation between network modularity and habitat variability was observed, as reported in the previous study [10]. This result indicates that the procedures used for data analysis in this study were not problematic, and it implies that the observed increase in network modularity due to habitat variability might result from a lack of data on metabolic reactions.

### Horizontal gene transfer also hardly explains the increase in metabolic network modularity

Horizontal gene transfer may be useful for environmental adaptation. In particular, it is believed that horizontal gene transfer contributes to the evolution of metabolic networks in response to changes in the environments [41]. Survival in a variety of environments is closely related to habitat variability. Using the data on the extent of horizontal gene transfer in bacteria [42], [43], in fact, we found a positive correlation between the extent of horizontal gene transfer and habitat variability (Spearman's rank correlation coefficient *r_s_* = 0.29 and *p* = 0.0044; *p* = 0.00075 using Kruskal-Wallis test; see also Table S1). In addition to this, it has been hypothesized that horizontal gene transfer accelerates gene clustering [44]. These previous studies imply a correlation between horizontal gene transfer and metabolic network modularity. Thus, we also investigated the relationship between horizontal gene transfer and metabolic network modularity.

**Figure 2:**
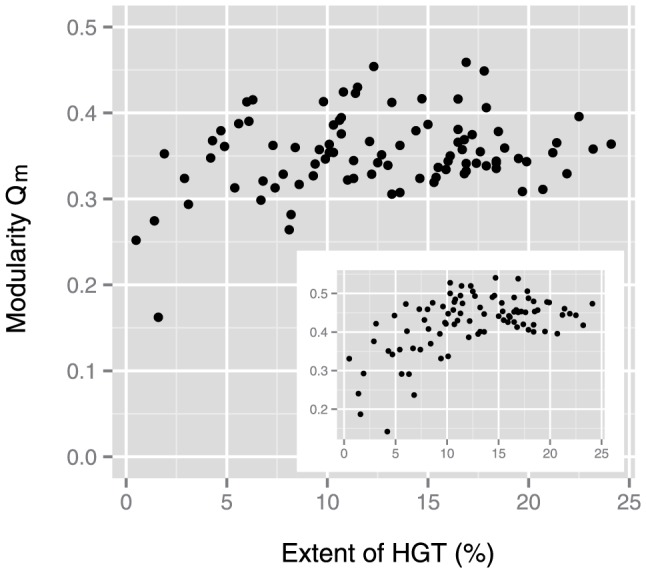
Correlation between the extent of horizontal gene transfer (HGT) and network modularity. Metabolic networks constructed from the latest version of the database show no correlation (Spearman's rank correlation coefficient *r_s_* = 0.15 and *p* = 0.15). Metabolic networks constructed from the early version of the database (inset) demonstrate a positive correlation (*r_s_* = 0.41 and the associated *p* = 4.8×10^−5^).

Originally, Kreimer et al. [42] demonstrated the positive correlation between the extent of horizontal gene transfer and network modularity in the context of gene clustering due to horizontal gene transfer; however, they used a different representation of metabolic networks compared to this study (i.e., enzymatic networks, in which nodes and edges represented enzymes and presence of interjacent chemical compounds, respectively). Using the data on horizontal gene transfer [42], [43] (see Table S1), we also re-confirmed such positive correlation (the inset of Figure 2) in the early version of metabolic networks.

In the latest version of metabolic networks, however, we could not conclude the positive correlation between the extent of horizontal gene transfer and metabolic network modularity (Figure 2). This result also suggests that the increase in network modularity because of horizontal gene transfer might be due to the lack of data on metabolic reactions.

A similar conclusion was derived in the context of enzymatic networks. As explained above, the impact of horizontal gene transfer on metabolic networks was concluded using enzymatic networks [42]. Using the latest version [28] and earlier version [39] of the metabolic reaction database, we constructed the latest version and early version of enzymatic networks on the basis of [45] (see Material and Methods for details) and evaluated the correlation between the extent of horizontal gene transfer and network modularity *Q*_m_.

In the enzymatic networks of the earlier version, the network modularity shows a positive correlation with the extent of horizontal gene transfer (*r_s_* = 0.37 and *p* = 0.00025). This result is in agreement with the conclusion derived by Kreimer et al. [42], although the normalization method of modularity is different between this study and the previous study. On the other hand, however, we could not conclude any correlation between network modularity and extent of horizontal gene transfer (*r_s_* = 0.12 and *p* = 0.24) in enzymatic networks of the latest version, as in the case of compound networks (Figure 2).

### Differences in structural properties between the earlier and latest versions of metabolic networks

We here discuss the effect of updates of the metabolic reaction database on network modularity. The database updates resulted in the metabolic networks of the latest version being larger than those of the earlier version. We considered the change ratios of the number of nodes *N* and edges *E*, where nodes and edges corresponded to metabolites and substrate–product relationships in metabolic reactions, respectively. The change ratio was defined as *R_X_* = *X*_latest_/*X*_earlier_−1, where *X*_latest_ and *X*_earlier_ are structural parameters (*N* or *E* in this case) in the latest and earlier versions of metabolic networks, respectively. This definition had a limitation in that it did not consider the loss of nodes and edges due to the database update; however, this discrepancy was minor–the latest metabolic network included approximately 98 and 95% of nodes and edges, respectively, contained in the earlier version.

**Figure 3:**
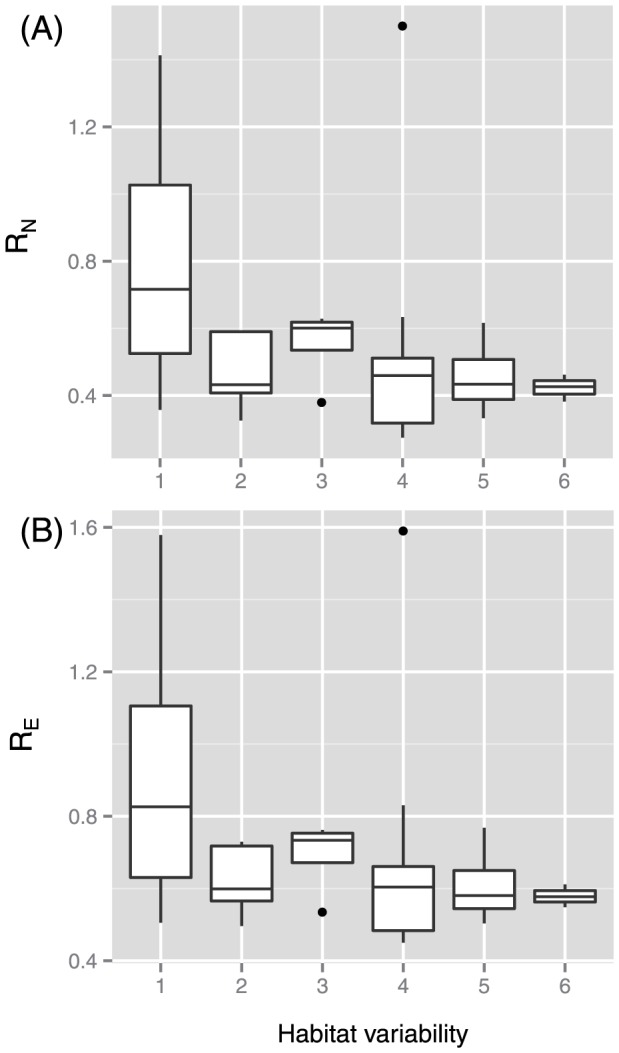
Change ratio of network parameters between the latest version and earlier version of metabolic networks. (A) In the case of the number of nodes (*p* = 1.5×10^−6^ using the Kruskal-Wallis (KW) test). (B) In the case of the number of edges (*p* = 8.8×10^−6^ using the KW test).

The mean and median *P_N_* in the metabolic networks of 115 bacteria were 0.59 and 0.48, respectively, while the mean and median of *P_E_* were 0.69 and 0.62, respectively. The median values of *P_N_* and *P_E_* were highest in species that showed the narrowest habitat variability (i.e., habitat variability of 1) (Figure 3). This result indicates that the metabolic networks of species with a habitat variability of 1 had been heavily updated.

**Figure 4:**
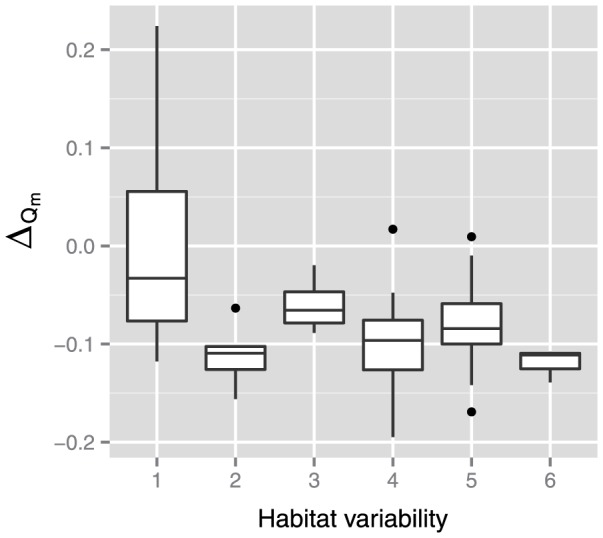
Differences in network modularity between the latest version and earlier version of the metabolic network database. The differences depend on habitat variability (*p* = 1.9×10^−7^ using the Kruskal-Wallis test).

In contrast, the normalized modularity values in the latest version were overall slightly smaller than those of the earlier version. The mean and median differences in modularity between the latest and earlier versions (Δ*_Qm_* = *Q_m_* ^latest^−*Q_m_* ^earlier^) were −0.07 (*p*<2.2×10^−16^, paired-sample *t*-test) and −0.08 (*p* = 3.0×10^−13^, paired-sample Wilcoxon signed rank test), respectively. In particular, the *Q_m_* value decreased significantly in species with higher habitat variability (Figure 4). This decline of *Q_m_* negates the positive correlation between *Q_m_* and habitat variability observed in the previous study [10].

**Figure 5:**
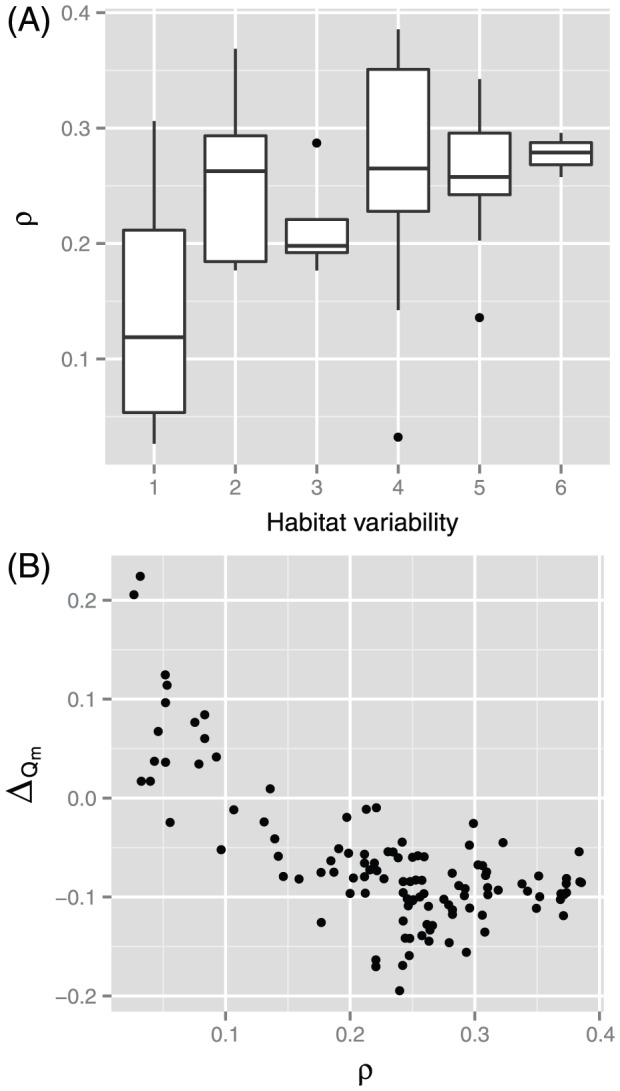
Ratio *ρ* of the number of new edges among nodes in the earlier version of the database to all newly added edges. (A) The ratio *ρ* depends on habitat variability (*p* = 6.8×10^−8^ using the Kruskal-Wallis test). (B) The ratio *ρ* negatively correlates with the difference Δ*_Qm_* (Spearman's rank correlation coefficient *r_s_* = −0.56 and *p* = 1.0×10^−10^).

However, the differences in *R_N_* and *R_E_* cannot simply explain the difference in *Q_m_* between the latest and earlier versions, although they indicate the degree of network expansion due to the database update. As mentioned in the previous section, the parameters *N* and *E* do not strongly affect *Q_m_*, as they were not correlated with this parameter due to normalization of the network modularity value. Therefore, it is difficult to clearly explain the change in *Q_m_*. However, *Q_m_* may have changed because the addition of new metabolic reactions differs between species with narrow and higher habitat variability. Here, we focused on the ratio (*ρ* of the number of new edges among nodes that existed in the earlier database to the total number of new edges due to the database update). The mean and median values of *ρ* were 0.21 and 0.23, respectively, and *ρ* was lowest in species with a habitat variability value of 1 (Figure 5A). In species with higher habitat variability, new metabolic links (i.e., substrate–product relationships) tended to be drawn among metabolites that existed in the earlier version of the database. In species with a habitat variability value of 1, new substrate–product relationships and metabolites (i.e., radically new metabolic pathways not found in the earlier database) tended to be added.

Taken together, we speculate that the change in *Q_m_* due to updating of the database occurred for the following reasons. In general, network modularity decreased slightly because metabolic networks may be randomized due to the addition of edges among previously existing nodes, although this does not imply that all edges contributed to the randomization. However, network modularity may remain constant or increase because of the addition of many new metabolic pathway modules in species with narrow habitat variability. This speculation may be supported by the negative correlation Δ*_Qm_* and *ρ* (Figure 5B).

### Metabolic network modularity may be influenced by growth conditions rather than by habitat variability

Because habitat variability did little to describe the increase in modularity (Figure 1), other explanations for the variation in modularity among species were investigated. Species' growth conditions correspond to their metabolic network structure. To evaluate network modularity as a function of species' growth conditions, we selected 383 bacterial species with identified growth conditions (oxygen requirement and growth temperature) from the list [14] (see also Table S1).

**Figure 6:**
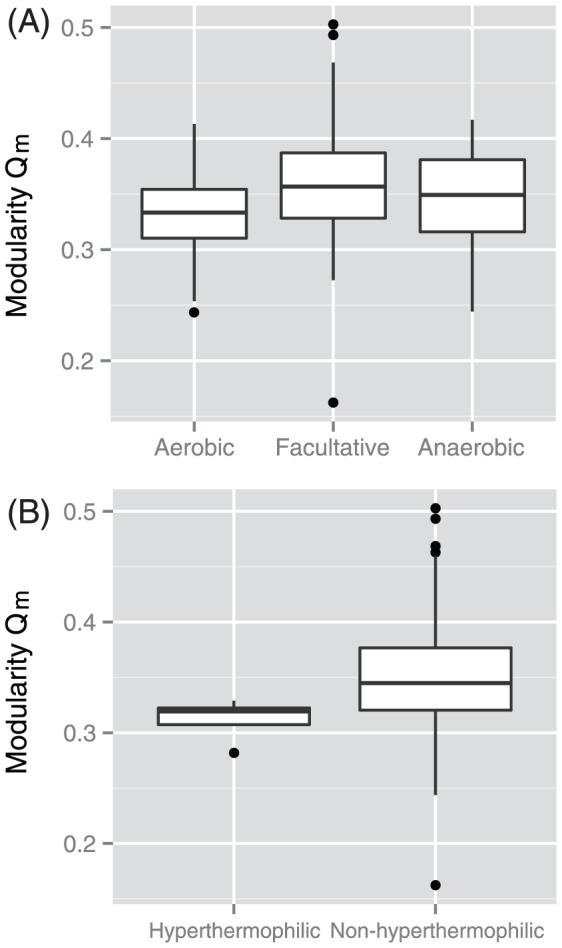
Differences in network modularity values due to differences in species' growth conditions. (A) Oxygen requirements (*p* = 8.7×10^−8^ using the Kruskal-Wallis test). The degree of oxygen required increases in the following order: anaerobic, facultative, and aerobic. (B) Growth temperature (*p* = 0.02 using the Wilcoxon test). Growth temperature increases from non-hyperthermophilic to hyperthermophilic.

We found differences in the network modularity of bacteria due to differences in growth conditions (Figure 6). In particular, the network modularity of aerobic bacteria was slightly lower than that of facultative and anaerobic bacteria. Moreover, hyperthermophilic bacteria showed lower metabolic network modularity than non-hyperthermophilic bacteria. This finding is consistent with the result shown by Kreimer et al. [42]; however, the representation of metabolic networks used by Kreimer et al. was different from that used in this study (i.e., enzymatic networks). Similar tendencies were also observed in archaeal metabolic networks [22]. Thus, differences in network modularity due to varying growth conditions such as temperature may be conserved in prokaryotes.

## Discussion

The results presented here call into question the impact of habitat variability and its closely related parameter (i.e., the extent of horizontal gene transfer) on metabolic network modularity (Figures 1 and 2). The positive correlation of network modularity with habitat variability and the extent of horizontal gene transfer shown in the previous study were probably due to a lack of data on metabolic reactions (i.e., questionable data accuracy). This correlation may be invalidated by the improved metabolic information that is now available for species with narrow habitat variability (or with low extents of horizontal gene transfer).

Figure 6 implies that network modularity is dependent on species' growth conditions, as in the case of Archaea [22]. For example, the lower metabolic network modularity observed in aerobic bacteria may be the result of incorporation of peripheral metabolic reactions due to oxygen [42], [46]. Furthermore, the thermal stability of proteins (metabolic enzymes) may yield lower metabolic network modularity observed in hyperthermophilic bacteria. Enzymes require a certain level of structural stability to survive in hot environments; thus, they tend to be easily deactivated (i.e., disappearance of edges) under such conditions, and as a result, the modular structure may collapse. The details of possible mechanisms underlying network modularity based on these growth conditions have been discussed previously [22], [27].

It may be suitable to conclude that the impact of growth conditions such as growth temperature on network modularity is limited in bacteria because the differences in network modularity are not very significant (Figure 6). This may be because bacteria have very similar growth conditions. Results from our theoretical study [27] and an additional previous study [47] suggest that metabolic network modularity can be acquired almost neutrally when there are no significant selective constraints. For example, temperature is a strong selective constraint [48]. Our theory predicts that network modularity differs little among species because most bacteria show similar growth temperatures (approximately 93% of the 383 bacteria that we investigated are mesophiles). On the other hand, the modularity in archaeal metabolic networks shows a strong dependency on growth conditions–temperature in particular–because Archaea are widely distributed among a wide range of environments [23]. Several theoretical studies [47], [49] have also reported the neutrality of structural properties in metabolic networks.

Zhou and Nakhleh [50] reported a similar conclusion. Inspired by our previous study [22], they showed the association between network modularity and these growth conditions on a large set of species spanning a wider range of taxonomy (i.e., Archaea, Bacteria, and Eukaryotes). This finding strongly supports our results, including our previous results [22], [24]. Zhou and Nakhleh also reported no correlation between habitat variability and metabolic network modularity; however, they could not clearly conclude the reason of the contradictory finding with the previous study. Zhou and Nakhleh discussed that the limited impact of habitat variability might be because of differences in network reconstruction, algorithm used to optimize modularity, or data used, and they did not give emphasis to this discrepancy. In this study, we showed that the hypothesis in which habitat variability promotes metabolic network modularity is highly likely to result from the accuracy of metabolic information, and emphasize that this hypothesis, which is accepted in the wide-ranging research fields such as molecular biology and ecology, is an open question.

These findings do not entirely discount the impact of habitat variability. Rather, they stress the necessity of a more suitable definition of habitat variability. Parter et al. [10] have defined habitat variability on the basis of an NCBI BioProject database (www.ncbi.nlm.nih.gov/bioproject). Although ecological or environmental relationships of cellular (especially metabolic) networks have been investigated on the basis of this definition (e.g., [11]–[14]), it may not correctly reflect real-world variability in natural habitats. The definition of habitat variability is more complicated than that of growth conditions; thus, careful examination of the interactions of metabolic network modularity with habitats and environments is required. To do this, it is necessary to capture habitat and environmental metadata. For example, the Environmental Ontology (EnvO) database [51] for concise and controlled descriptions of environments may provide more detailed definitions through hierarchical classification schemes.

The impact on horizontal gene transfer is also an open question. The identification of horizontally transferred genes is difficult. The extent of horizontal gene transfer, used in this study and previous studies, is limited to the recent events of horizontal transfer because of the sensitivity of the method [43]. When the time scale of the events is extended, we may be able to conclude a positive correlation between the extent of horizontal gene transfer and network modularity. In particular, Cordero and Paulien [52] found a surprising pattern of nonlinear enrichment of long-distance horizontal gene transfers in large genomes focusing on both cumulative and recent evolutionary histories, suggesting that distant horizontal transfers are biased toward specific functional groups. Characterization of the difference in enrichment patterns between recent and cumulative horizontal gene transfers may reveal the impact of horizontal gene transfer on metabolic network modularity in greater detail. In particular, Cordero and Paulien suggested an intimate relationship between environmental and genomic complexity in microbes, which implies that an ecological, as opposed to phylogenetic, signal in gene content is relatively important in bacteria. This indication may be important to understand the relationship between several factors (e.g., habitat variability, growth conditions, and horizontal gene transfers) believed to interact with metabolic network modularity.

The definition of modularity and modules is also controvertible. The conclusion in this study is limited in the context of network modularity. For metabolic networks, however, most biologically functional modules may be hardly defined through module detection methods based on network topology (i.e., in the context of network modularity). In particular, it is reported that the definition of modularity, used in this study and many previous studies, might not be topologically intuitive due to the locality and limited resolution [53], [54]. Methods based on link communities (e.g., [55], [56]) may be useful to avoid these limitations because they show better accuracy in the prediction of biologically functional modules (or categories such as pathways).

It is also necessary to test the effect of growth conditions such as temperature on network modularity using more species, although current data on species phenotypes are biased as mentioned above. Therefore, it is important to identify species that live in extreme environments (i.e., extremophiles). The development of high-throughput techniques may provide more such data. For example, metagenomics using next-generation sequencing helps to identify novel extremophiles from hot springs, deep sea, and so on. By considering more extremophiles, the effect of growth conditions on metabolic network modularity could be more appropriately evaluated.

Metabolic networks have not been fully understood; thus, there is a need for a more careful examination in data analysis in the future. For example, enzyme promiscuity [57], which implies that enzymes can catalyze multiple reactions, act on more than one substrate, or exert a range of suppressions [58], in which an enzymatic function is suppressed by over-expressing enzymes showing originally different functions, suggests the existence of many hidden metabolic reactions, and they may be related to metabolic robustness against changing environments [59]. Consideration of these hidden metabolic reactions is important for designing metabolic pathways and understanding metabolic evolution.

Our analysis has several limitations, as do many other works on metabolic network analyses: limited knowledge of metabolic reactions (i.e., missing links), reconstruction of metabolic networks based on genomic information, and failure to consider reaction stoichiometry and direction of reaction (i.e., reversible or irreversible).

Although data analysis has these limitations, these findings encourage a reconsideration of the widely accepted hypothesis (i.e., the impact of habitat variability on metabolic network modularity), and they enhance our understanding of adaptive and evolutionary mechanisms in metabolic networks.

## Materials and Methods

### Construction of metabolic networks

We downloaded XML files (version 0.7.1) containing the metabolic network data on May 20, 2011 from the KEGG database [14] (ftp://ftp.genome.jp/pub/kegg/xml/kgml/metabolic/organisms/). As of July 1, 2011, the KEGG FTP site is only available to paid subscribers. Since the use of such data may be desirable to ensure reproducibility, our dataset on metabolic networks is available upon request. Instead of the KEGG databases, we can consider other databases and datasets such as MetaCyc [15] and biochemical reaction databases of the Institute of Bioprocess and Biosystems Engineering [28], [39].

These metabolic networks are represented by undirected networks (i.e., compound networks) in which nodes and edges correspond to metabolites and reactions (i.e., substrate–product relationships), respectively. We extracted R numbers (e.g., R00010), which indicate metabolic reactions, from the XML files. On the basis of R numbers, substrate–product relationships were identified as carbon traces using the latest version [28] and an earlier version [39] of the metabolic reaction database. Currency (ubiquitous) metabolites such as H_2_O, ATP, and NADH were removed as described previously [24]. The largest connected component (giant component) was extracted from each metabolic network to accurately calculate network modularity (and also for comparison with the previous study [10]). In particular, network randomization (in the calculation of *Q*_m_ in this study) requires caution in case the networks possess isolated components or (clusters) [60] because *Q*_m_ may be overestimated or underestimated due to isolated components; thus, we avoided the use of entire networks.

Using these datasets, for species in which the extent horizontal gene transfer has been determined in [43], we constructed enzymatic networks, in which the nodes and edges are metabolic enzymes (reactions) and presence of interjacent chemical compounds, respectively. Basically, an edge is drawn between 2 enzymes (nodes) if at least 1 product of a reaction catalyzed by an enzyme corresponds to at least 1 substrate of the reaction catalyzed by another enzyme (see [3], [45] for details). Substrate–product relationships of each metabolic reaction were defined as carbon traces using the latest version [28] and an earlier version [39] of the metabolic reaction database in order to avoid the emergence of biologically unsuitable edges (see [3], [45] for details; the importance of this handling is explained with an example). The largest connected component (giant component) was extracted from each metabolic network to avoid bias from small isolated components in the calculation of *Q_m_*.

### Measurement of metabolic network modularity

This method is similar to that of a previous study [10], thereby allowing comparison.

To allow the comparison of metabolic network modularity with networks of different size and connectivity, we used the normalized network modularity value *Q_m_* based on [10], [22], [27], which was defined as:
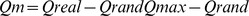



where *Q*_real_ is the network modularity of a real-world metabolic network and *Q*_rand_ is the average network modularity value obtained from 10000 randomized networks constructed from its real-world metabolic network. The network modularity measure *Q* is defined as the fraction of edges that lie within, rather than between, modules relative to that expected by chance (see equation (4) in [32]). Each *Q* value was calculated using the fast greedy algorithm proposed by Clauset et al. [32]. *Q*_max_ was estimated as: 1−1/*M*, where *M* is the number of modules in the real network.

Randomized networks were generated from a real-world metabolic network using the edge-rewiring algorithm [60]. This algorithm generates a random network by rewiring 2 randomly selected edges until the rewiring of all edges is completed. For example, consider 2 edges, A−B and C−D, where the letters and lines are nodes and edges, respectively. Through this edge-rewiring algorithm, the edges A−D and C−B are obtained (see [60] for details).

In general, in metabolic networks (i.e., substrate graphs) where reactions have multiple substrates and products, short cycles related to network modularity are generated as a result of network representations [22]. Ideally, the number of short cycles should remain constant during the generation of randomized networks. However, the edge-rewiring algorithm used here does not abide by this constraint. Although the null model has this limitation, it did not pose a significant problem in this study because the substrate graphs used were based on atomic mapping and currency metabolites were excluded. As an example, we considered a straight-line pathway, which is a part of the central metabolism (see Figure S1A). In the case of substrate graphs drawn according to chemical equations, in which a metabolic reaction A+B → C+D is converted to a graph with 4 edges: A−B, A−C, B−C, and B−D, short cycles were drawn to some of the edges connecting to currency metabolites (ATP and ADP in this case) despite the straight-line metabolic pathway (Figure S1B). When focusing on atomic traces (carbon traces, in particular) and removing currency metabolites, such short cycles are not drawn (Figures S1C and S1D). Hence, in our metabolic networks, most of the metabolic reactions (approximately 96% on average) are represented as reactions with a single substrate and/or product. Therefore, short cycles generated by network representation rarely pose problems.

## Supporting Information

Figure S1 **Short cycles drawn according to network representations.** (A) A representation of a metabolic pathway as depicted in textbooks. Examples of substrate graphs based on chemical equations (B) and atomic traces (C). The solid and dashed lines represent traces based on carbon and phosphorus atoms, respectively. (D) Substrate graph based on atomic traces after removal of currency (ubiquitous) metabolites. The direction of edges is omitted in (B–D).
(PDF)

Table S1 **List of bacterial species.** This table includes the species name, Kyoto Encyclopedia of Genes and Genomes (KEGG) ID (see http://www.genome.jp/kegg/catalog/org_list.html), genome size, number of protein-encoding genes, habitat variability, oxygen requirements, growth temperature, and extent of horizontal gene transfer for each bacterium. In addition, it includes the following parameters from the latest and earlier versions of each bacterial metabolic network (compound network): modularity value *Q*, normalized modularity value *Q_m_*, number of nodes (metabolites), number of edges (substrate–product pairs), and number of new edges among nodes that existed in the earlier version. In the case of the latest version of metabolic networks, the modularity value *Q*_SA_ in the largest connected component calculated using the simulated annealing-based method and the modularity value *Q*_Entire_ in the entire networks calculated using the fast greedy algorithm are also provided. In addition to this, this table also includes the network parameters of enzymatic networks: *Q*, *Q*_m_, number of nodes (enzymes), and number of edges (presence of shared metabolites).
(XLS)
