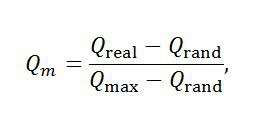# Correction: Does Habitat Variability Really Promote Metabolic Network Modularity?

**DOI:** 10.1371/annotation/80e66eeb-6dd3-4117-8bbd-7d3c7d692a5c

**Published:** 2013-10-24

**Authors:** Kazuhiro Takemoto

An error was introduced in the preparation of this article for publication. The standalone equation in the Methods section under the subheading “Measurement of metabolic network modularity” is incorrect. The correct equation is: