# Supplementary figures and images for: Does Habitat Variability Really Promote Metabolic Network Modularity?
Source: PLoS One. 2013 Apr 12;8(4):e61348. doi: 10.1371/journal.pone.0061348 (PMC3625173; doi:10.1371/journal.pone.0061348)

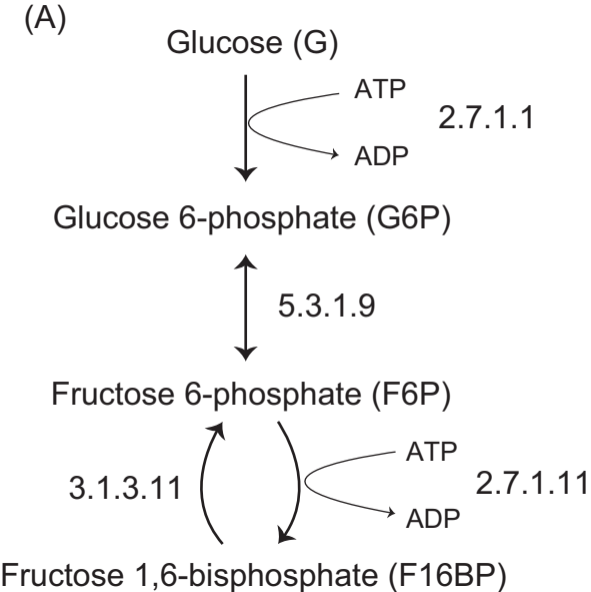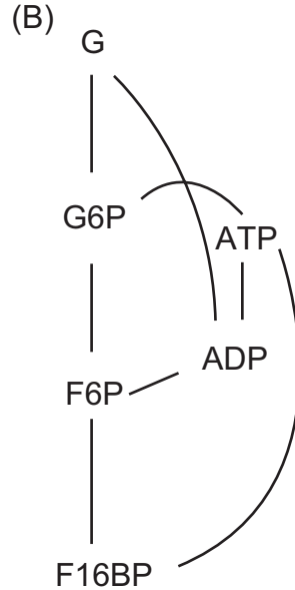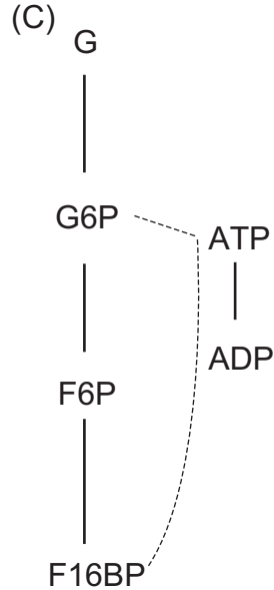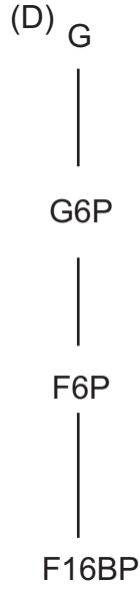

Supplement: Figure S1 — Short cycles drawn according to network representations. (A) A representation of a metabolic pathway as depicted in textbooks. Examples of substrate graphs based on chemical equations (B) and atomic traces (C). The solid and dashed lines represent traces based on carbon and phosphorus atoms, respectively. (D) Substrate graph based on atomic traces after removal of currency (ubiquitous) metabolites. The direction of edges is omitted in (B–D). (PDF) [file pone.0061348.s001.pdf]
